# Retrospective validation of the postnatal Growth and Retinopathy of Prematurity (G-ROP) criteria in a Swiss cohort

**DOI:** 10.1186/s12886-021-02227-4

**Published:** 2022-01-10

**Authors:** Nithursa Vinayahalingam, Jane McDougall, Olaf Ahrens, Andreas Ebneter

**Affiliations:** 1 Department of Ophthalmology, Inselspital, Bern University Hospital, University of Bern, Bern, Switzerland; 2 Department of Neonatalogy, Inselspital, Bern University Hospital, University of Bern, Bern, Switzerland

**Keywords:** Screening, Retinopathy of Prematurity, G-ROP, Swiss Cohort

## Abstract

**Background:**

Currently used screening criteria for retinopathy of prematurity (ROP) show high sensitivity for predicting treatment-requiring ROP but low specificity; over 90% of examined infants do not develop ROP that requires treatment (type 1 ROP). A novel weight gain-based prediction model was developed by the G-ROP study group to increase the specificity of the screening criteria and keep the number of ophthalmic examinations as low as possible. This retrospective cohort study aimed to externally validate the G-ROP screening criteria in a Swiss cohort.

**Methods:**

Data from 645 preterm infants in ROP screening at Inselspital Bern between January 2015 and December 2019 were retrospectively retrieved from the screening log and analysed. The G-ROP screening criteria, consisting of 6 trigger parameters, were applied in infants with complete data. To determine the performance of the G-ROP prediction model for treatment-requiring ROP, sensitivity and specificity were calculated.

**Results:**

Complete data were available for 322 infants who were included in the analysis. None of the excluded infants had developed type 1 ROP. By applying the 6 criteria in the G-ROP model, 214 infants were flagged to undergo screening: among these, 14 developed type 1 ROP, 9 developed type 2 ROP, and 43 developed milder stages of ROP. The sensitivity for predicting treatment-requiring ROP was 100% (CI, 0.79–1.00), and the specificity was 41% (CI, 0.35 –0.47). Implementing the novel G-ROP screening criteria would reduce the number of infants entering ROP screening by approximately one third.

**Conclusions:**

The overall prevalence of treatment-requiring ROP was low (2.15%). Previously published performance parameters for the G-ROP algorithm were reproducible in this Swiss cohort. Importantly, all treatment-requiring infants were correctly identified. By using these novel criteria, the burden of screening examinations could be significantly reduced.

## Background

Retinopathy of prematurity (ROP) is one of the leading preventable causes of childhood vision impairment in high income countries [1]. To prevent unfavourable outcomes of ROP, all infants at our institution with birthweight (BW) < 1500 g and gestational age (GA) < 32 weeks undergo repeated ophthalmic examinations according to the UK retinopathy of prematurity screening guidelines [2]. Even though many extremely preterm infants develop some degree of ROP, most of them do not require any treatment. In fact, less than 10% of examined infants with the current screening criteria develop a treatment requiring ROP (type 1 ROP). Therefore, the vast majority of ophthalmic examinations are performed in children who never need an intervention [3].

To minimize the burden of screening, alternative criteria have been proposed by several groups [3–9]. One of the most promising algorithms, the G-ROP model, was developed using a large database [10, 11]. It is based on the assessment of 6 trigger criteria: gestational age (GA) less than 28 weeks, birth weight (BW) less than 1051 g, weight gain less than 120 g during 10 to 19 days postnatal age (PNA), weight gain less than 180 g during 20 to 29 days PNA, weight gain less than 170 g during 30 to 39 days PNA, or hydrocephalus. The G-ROP model has been validated in several populations, [12–14] which is crucial since characteristics of premature infants vary depending on their socioeconomic environment [15].

To validate the novel G-ROP prediction model externally in a Swiss cohort and to determine the performance, the G-ROP screening criteria were retrospectively tested in a representative local cohort.

## Methods

This retrospective observational study was approved by the local ethics committee Kantonale Ethikkommission Bern (KEK 2020-01064) and was performed in accordance with the Declaration of Helsinki and relevant guidelines and regulations. Written informed consent was waived by the ethics committee. The data were stored in deidentified format in a REDCap® database hosted by the Clinical Trials Unit of the Faculty of Medicine of the University of Bern and the Inselspital, Bern University Hospital.

All data were retrospectively retrieved at Inselspital Bern University Hospital, which is one of the eight Swiss hospitals with level 3 neonatal intensive care. In the catchment area, the prevailing ethnicity is Caucasian, and approximately 14,500 children are born per year. On average, 168 newborns below 32 weeks GA or below 1501 g BW are hospitalized in this unit [16].

Data from preterm infants (GA < 37 weeks) who underwent retinal screening examinations for ROP between January 2015 and December 2019 were collected and analysed. As per the local hospital policy, the UK screening guidelines are followed to determine the need for newborns to enter ROP screening [2]. Moreover, preterm infants with BW < 2000 g and any type of oxygen supplementation additionally entered screening according to local guidelines. After uneventful clinical course and discharge from our tertiary institution, all infants were regularly followed up in peripheral hospitals or private practices according to UK guidelines, if needed [2].

ROP outcomes were retrospectively retrieved from the neonatal medical records, along with the sequential weight measurements, infants’ GA, BW, sex, and hydrocephalus status. ROP outcomes, representing the most advanced stage reached in the course of the disease, were classified into type 1 ROP, type 2 ROP, and low-grade ROP. Low-grade ROP was defined by mild retinal changes outside the range of normal development but not severe enough to meet criteria for type 2 ROP. Data from infants with an incomplete dataset were excluded from the final analysis. Importantly, none of the infants with missing data developed type 1 ROP.

For the infants with a complete data set, the six criteria of the G-ROP prediction model (gestational age (GA) less than 28 weeks, birth weight (BW) less than 1051 g, weight gain less than 120 g during 10 to 19 days PNA, weight gain less than 180 g during 20 to 29 days PNA, weight gain less than 170 g during 30 to 39 days PNA or hydrocephalus) were applied. As soon as any of these parameters are met in an infant, it qualifies for ROP screening according to this algorithm. When an infant does not meet any of the 6 criteria, retinal ROP examinations are not warranted according to the G-ROP model [11].

The performance of the G-ROP prediction paradigm was determined by calculating the sensitivity and specificity for predicting type 1 ROP. Additionally, 95% confidence intervals were calculated by using the Wilson score method.

## Results

**Fig. 1:**
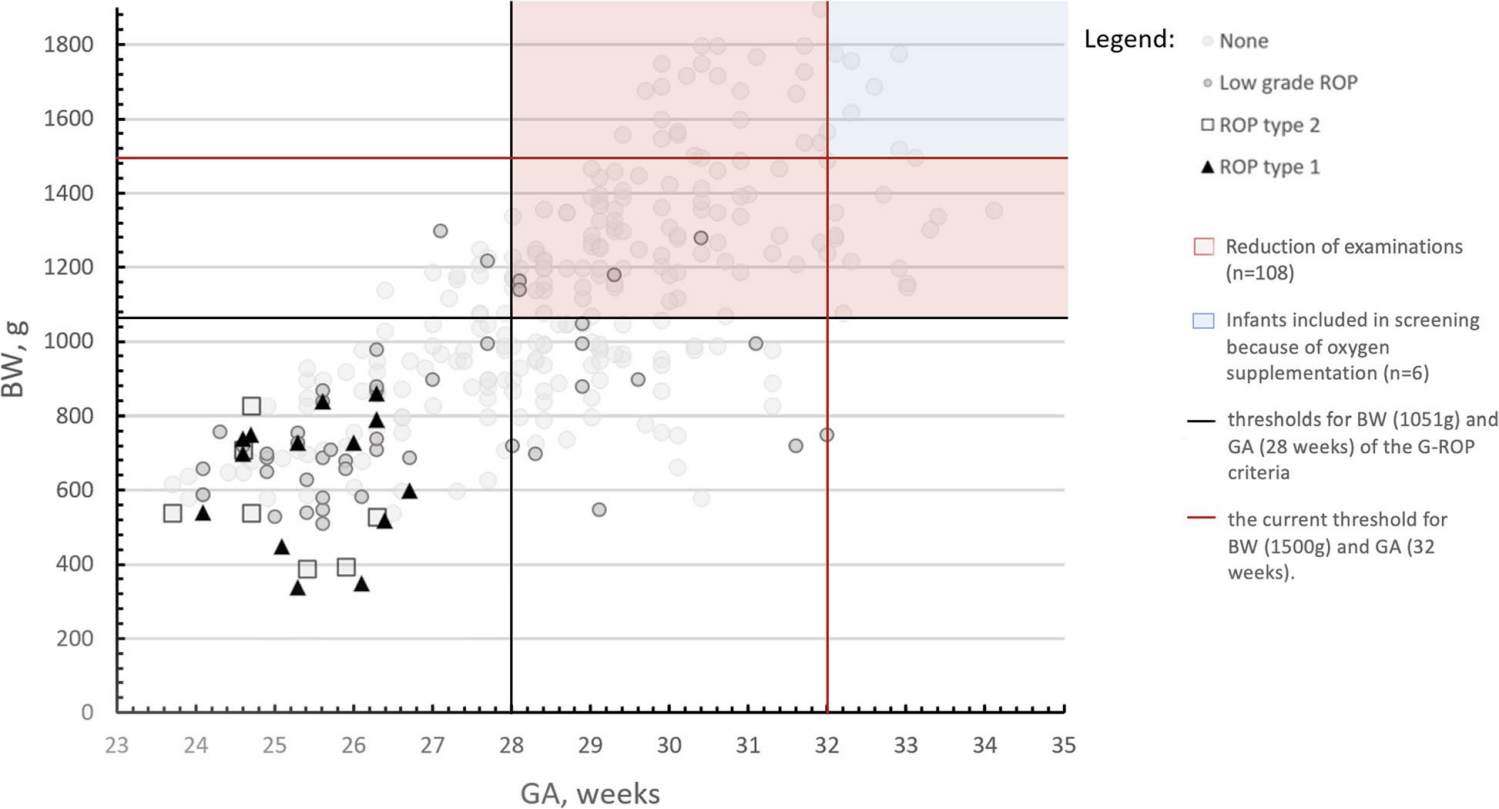
Scatter plot illustrating the effect of the G-ROP criteria on screening indication. Each study subject is represented by its gestational age (GA) and birth weight (BW) in this graph. The currently used thresholds for ROP screening inclusion are indicated by the red lines. The thresholds for GA and BW in the G-ROP criteria are shown by the black lines. All infants in the area hatched in red would not have qualified for ROP screening using the G-ROP criteria. As per local guidelines, all preterm infants (GA < 37 weeks) with oxygen supplementation also enter ROP screening regardless of BW. These infants (*n* = 6) are represented in the upper right corner of the graph in the light blue hatched area. None of these children developed ROP. The clinical outcome related to ROP is represented by the shape of the individual data point. Infants represented by black triangles developed type 1 ROP, those represented by squares type 2 ROP, and those represented by dark circles low-grade ROP. Infants shown by the light circles did not have clinical evidence for any ROP in serial fundus examinations

In total, 645 preterm infants who underwent retinal examinations between 30–37 PMA were identified. For 322 of them, the complete dataset was available, and they were included in the final analysis. Seventy infants developed ROP: 14 developed ROP type 1, 9 developed ROP type 2, and 47 developed low-grade ROP (Fig. 1). The characteristics of these children are summarized in Table 1. The median BW in this cohort was 1050 g, and the median GA was 28.4 weeks. A total of 323 infants with incomplete information about weight gain were excluded from the study. Among the excluded infants, the median BW was 1490 g, and the median GA was 32.3 weeks. Nine excluded children developed low-grade ROP not requiring an intervention. Of note, none of them developed type 1 ROP.

**Table 1:** Characteristic of Infants

	**ROP type 1**	**ROP type 2**	**Low-grade**	**None**	**Excluded**
**Birthweight, g**					
** mean (SD)**	637 (167)	596 (145)	799 (209)	1134 (301)	1588 (498)
** median**	705	540	720	1145	1490
**gestational age, weeks**					
** mean (SD)**	25.5 (0.8)	25.1 (0.8)	26.8 (2.0)	28.9 (2.1)	32.5 (2.6)
** median**	25.4	24.7	26.3	29	32.3
** male, n (%)**	7 (50)	4 (44.4)	25 (53.2)	136(53.9)	186(53.0)

By applying the G-ROP model, an alarm was triggered in 215 children (Table 2). All infants with treatment requiring ROP were identified by the two parameters BW < 1051 g and/or GA < 28 weeks. Thus, the G-ROP model raised alarms correctly for all 14 ROP type 1 children, all 9 children with ROP type 2, and 43 of 47 children with low-grade ROP. The 4 children who were not flagged by the G-ROP algorithm developed low-grade ROP only. All of them had additional risk factors for ROP: necrotising enterocolitis, patent ductus arteriosus and prolonged oxygen supplementation with or without the diagnosis of bronchopulmonary dysplasia [11].

**Table 2:** Prediction of ROP by the G-ROP screening criteria and characteristics of infants

	**Alarm + **	**Alarm -**	**Total**	**Sensitivity**	**Specificity**
**ROP type 1, n**	14	0	14	100%	
**ROP type 2, n**	9	0	9	100%	
**Low-grade, n**	43	4	47	91.5%	
**None, n**	148	104	252		41.3%
**Total, n**	214	108	322		

The sensitivity of the G-ROP criteria for predicting treatment requiring ROP was 100% (CI, 0.79–1.00), and the specificity was 41% (CI, 0.35 –0.47) in this cohort. Consequently, 108 infants less would have entered screening with the G-ROP algorithm, compared to currently used screening criteria [2] four of them eventually developed low-grade ROP, but none of them type 1 or type 2 ROP. This suggests that in this cohort, the G-ROP algorithm would have safely reduced the indication for ROP screening by 33%.

## Discussion

All infants with type 1 and type 2 ROP were correctly identified by the G-ROP model. Four children with low-grade ROP were not flagged by the G-ROP algorithm and would not have entered screening. Interestingly, according to medical records, these infants all showed additional risk factors, which are associated with ROP. Nevertheless, the sensitivity for predicting treatment requiring ROP was 100%, and the sensitivity for low-grade ROP was 91%. Implementing the new G-ROP screening criteria in daily clinical practice would reduce the number of screened infants by approximately one third. Similar results were found in other validation studies for the G-ROP algorithm [12–14].

Larsen et al. have suggested that modifying the GA threshold to < 30 weeks in the screening guidelines would lower the burden of screening examinations with acceptable impact on the sensitivity of screening programs [3]. Indeed, applying this modified GA limit of < 30 weeks would not have altered the screening sensitivity for predicting treatment-required ROP in this cohort. These findings indicate that the current screening guidelines could be re-evaluated and potentially be optimized. However, even in highly developed healthcare systems, increasing the threshold for entering ROP screening carries the risk of failure to detect infants who require sight-saving interventions.

There are some limitations to consider. Some of them are not limited to this study, but more generally apply to ROP screening criteria in any clinical settings: The influence of the socioeconomic context does not allow generalization of findings. While in some high-income countries the sensitivity of G-ROP criteria to predict type 1 ROP in validation studies was 100%, [11, 12] lower sensitivity (91.9%) has been suggested in less mature healthcare systems such as Turkey [13]. These differences may have resulted from different neonatal care practices and different characteristics of the premature population in low- and middle-income countries [15].In Switzerland and Germany, less than 1% of infants with GA > 30 weeks developed treatment requiring ROP, [3, 17] but in Mexico, over one-third of preterm infants with GA > 32 weeks may develop ROP type 1 [18]. Moreover, careful attention is needed in countries with less tightly controlled use of oxygen supplementation, as postnatal weight gain is not a reliable predictive factor of ROP when an infant is treated with excessive oxygen supplementation [14]. It is therefore extremely important to validate any new criteria in a population before adopting them.

Limitations specific to this study are the retrospective design and the single centre setup. Moreover, since data were recorded at a tertiary healthcare centre with a policy to transfer patients to peripheral hospitals close to their home as timely as medically possible, many infants had incomplete data and were excluded from the final analysis. However, according to a post-hoc sensitivity analysis, the sensitivity for predicting type 1 ROP was the same including all 645 children. In the present setting, the likelihood of missing treatment requiring disease is very low, since Inselspital Bern University Hospital is the only centre in our region that provides treatment for ROP.

## Conclusions

In conclusion, the overall prevalence of treatment requiring ROP was low (2.15%). Previously reported performance parameters for the G-ROP algorithm were reproducible in this Swiss cohort. Importantly, all treatment requiring infants were correctly identified. By using these novel criteria, the number of infants entering ROP screening could be reduced by approximately one third. These findings suggest that the current screening guidelines could be optimised, and that the G-ROP algorithm seems to be a suitable starting point to be evaluated in a larger population.

## Data Availability

The datasets generated and/or analyzed during the current study are not publicly available due to privacy concerns but are available from the corresponding author on reasonable request.
